# Changing nutrition care practices in hospital: a thematic analysis of hospital staff perspectives

**DOI:** 10.1186/s12913-017-2409-7

**Published:** 2017-07-19

**Authors:** Celia Laur, Renata Valaitis, Jack Bell, Heather Keller

**Affiliations:** 1 Department of Applied Health Science, University of Waterloo, 200 University Ave, Waterloo, ON N2L 3G1 Canada; 2 School of Human Movement and Nutrition Sciences, The University of Queensland &, The Prince Charles Hospital, Rode Road, Chermside, QLD 4032 Australia; 3 Schlegel-University of Waterloo Research Institute for Aging, Waterloo, ON Canada; 4 Department of Kinesiology University of Waterloo, 200 University Ave, Waterloo, N2L 3G1 ON Canada

**Keywords:** Hospital, Malnutrition, Staff opinions, Making change, Implementation

## Abstract

**Background:**

Many patients are admitted to hospital and are already malnourished. Gaps in practice have identified that care processes for these patients can be improved. Hospital staff, including management, needs to work towards optimizing nutrition care in hospitals to improve the prevention, detection and treatment of malnutrition. The objective of this study was to understand how staff members perceived and described the necessary ingredients to support change efforts required to improve nutrition care in their hospital.

**Methods:**

A qualitative study was conducted using purposive sampling techniques to recruit participants for focus groups (FG) (*n* = 11) and key informant interviews (*n* = 40) with a variety of hospital staff and management. Discussions based on a semi-structured schedule were conducted at five diverse hospitals from four provinces in Canada as part of the More-2-Eat implementation project. One researcher conducted 2-day site visits over a two-month period to complete all interviews and FGs. Interviews were transcribed verbatim while key points and quotes were taken from FGs. Transcripts were coded line-by-line with initial thematic analysis completed by the primary author. Other authors (*n* = 3) confirmed the themes by reviewing a subset of transcripts and the draft themes. Themes were then refined and further detailed. Member checking of site summaries was completed with site champions.

**Results:**

Participants (*n* = 133) included nurses, physicians, food service workers, dietitians, and hospital management, among others. Discussion regarding ways to improve nutrition care in each specific site facilitated the thought process during FG and interviews. Five main themes were identified: building a reason to change; involving relevant people in the change process; embedding change into current practice; accounting for climate; and building strong relationships within the hospital team.

**Conclusions:**

Hospital staff need a reason to change their nutrition care practices and a significant change driver is perceived and experienced benefit to the patient. Participants described key ingredients to support successful change and specifically engaging the interdisciplinary team to effect sustainable improvements in nutrition care.

**Trial registration:**

Retrospectively registered ClinicalTrials.gov Identifier: NCT02800304, June 7, 2016.

## Background

Globally, many studies have examined the prevalence of malnutrition [1–5], the barriers to food intake [6–8], and ways to protect mealtimes [9–12] in hospital. Few studies have attempted to describe how to improve hospital nutrition care practices and embed those practices in the unit routine [13]. An interdisciplinary approach is needed to improve the prevention, detection and treatment of hospital malnutrition [14, 15]. A key component of changing practice is to understand the views of those who will be involved in the change and the context or climate where the changes are occurring [16, 17]. Qualitative methods, including focus groups (FG) and key informant (KI) interviews, allow for this in-depth understanding [18].

The More-2-Eat (M2E) implementation project aims to optimize nutrition care in hospital through use of the Integrated Nutrition Pathway for Acute Care (INPAC) [19]. INPAC is an algorithm that recommends use of simple screening and assessment tools to diagnose malnutrition. Identification of barriers to food intake and food monitoring are also key activities to prevent iatrogenic malnutrition. Providing standardized advanced care strategies (e.g. oral nutrition supplementation) supports efficiently treating patients, and discharge planning is considered. As part of the M2E project, five hospitals in Canada are changing their nutrition care processes to align with INPAC. M2E is focused on developing and understanding the methods required for embedding the knowledge of INPAC into the routine of the unit [20]. A variety of methods are used throughout the M2E developmental (May-Dec 2015), implementation (Jan-Dec 2016), and sustainability phases (Jan-Mar 2017), to conduct process and outcome evaluation. All methods are described in a prior publication [20].

The M2E project is based on the action portion of the Knowledge-to-Action [21] cycle [22] and includes steps to understand context as well as barriers and facilitators to support change processes, and adoption of knowledge in order to promote sustainability. This qualitative study was designed to address these steps in the action cycle, but also aims to increase our understanding of what is necessary for implementing changes to nutrition care practices in hospital. A pragmatic approach was taken throughout the developmental phase of M2E due to the need to promptly understand context, as well as the barriers and facilitators to change required for sites to progress with their implementation efforts.

## Methods

### Overview

This was a qualitative descriptive study using thematic analysis with data collected at five diverse M2E hospitals, including: Royal Alexandra Hospital; Niagara Health, Greater Niagara General Site; The Ottawa Hospital; Concordia Hospital; and Pasqua Hospital Regina Qu’Appelle Health Region. Details of the sites are available elsewhere [20].

### Sampling and recruitment

FG (*n* = 11) and KI interviews (*n* = 40) were conducted during two-day site visits by CL in October/November 2015 at each site. A total of *n* = 133 participants were involved. Two FGs with 4-15 participants per group and 5-14 individual interviews were completed at each site. Despite evidence of similar issues being discussed by the time the third site was completed, all arranged site FG and interviews were completed to provide context-specific data to support implementation efforts. For the interviews, purposive sampling methods were used to select KIs to participate based on the criteria that they would provide valuable insight, both positive and negative, about nutrition care and making change on the unit or in the hospital [23]. For the FG, all staff on the M2E unit were invited; a minimum of two FG were scheduled for each site to capture staff on varying shifts. M2E champions and research associates, who led the implementation process at their hospital, conducted this recruitment using posters, e-mails and verbal reminders.

### Data collection

All interviews were conducted by CL, which increased credibility of results, as learnings and understandings built from interviews to FG and from site to site. CL is a female researcher and PhD candidate in health studies, with a background in public health nutrition and implementation science. She is not a health professional and not associated with any of the hospitals. CL did not meet participants before the site visits, however, before the discussions began, CL described her background to participants as well as the reason for the interview/FG.

During the FG and interviews, the environment (meeting room in the hospital) was made to feel comfortable, with a free lunch provided for FG discussions. Upon arrival, participants read and signed a consent form and completed a short demographic form. Each FG and interview took between 10 and 50 min and was digitally recorded. A M2E champion or research associate was in the room during the FG to take notes, and this was explained to the group and included in consent. When interview participants were not available during the 2-day visit, the interview was conducted by phone (*n* = 7). The discussions were based on a semi-structured guide (Table 1) that was adapted by CL during the interview, based on profession/role of the interviewee. The Holstein and Gubrium (1995) approach of Active Interviewing was used as it encourages the development of new questions based on interviewee responses allowing for the making of new connections and insights [24]. Context memos for each site were written by CL to elaborate on key observations and reflections at the end of the two-day site visit. This reflection process included reviewing audio-files and making preliminary summarizations of key data to be transferred to sites for consideration in their implementation process. As a first level form of member checking, each site was requested to respond to the summary if they did not feel it was an accurate representation.

**Table 1:** Guide for focus group and interview questions

Focus Group/Interview Questions Guide
1. What do you think this unit does well in terms of nutritional care?
2. What are the major challenges to providing nutrition care in this hospital?
3. In INPAC, we have suggested screening patients at admission by asking them 2 questions about weight change and food intake. What would help to make this change? What might prevent this change?
4. We want all patients to receive standard care, such as having packages opened, being set up to eat and ensuring that all patients have adequate access to food. What would help to make this change? What might prevent this change?
5. How can food intake of a patient be monitored? What would help to make this change? What might prevent this change?
6. *For RDs* – Are you familiar with SGA? Have you been trained? If SGA were to be done for all patients who are screened as at risk, what would help make this change? What might prevent this change?
7. If there was one thing you could change about the way food and nutrition care is provided on this unit, what would it be?
8. When you have made changes to improve care practices in the past, what worked well? What didn’t? Why?

*CNST* Canadian Nutrition Screening Tool, *RD* Registered Dietitian, *SGA* Subjective Global Assessment, *INPAC* Integrated Nutrition Pathway for Acute Care

Not all questions were asked of all participants and not all questions asked are listed here

All interview audio files were transcribed verbatim by a professional transcription service. FG recordings were not sent for transcription due to the volume of KI data and as a result, FG data were considered complementary in the analysis. Key points and quotes from each FG were obtained by listening to recordings a minimum of twice (CL).

### Data analysis

One researcher (CL) completed all initial analyses of interview transcripts, FG notes and context memos using NVivo 11 to support the coding structure and summarization of codes. Analysis followed the Saldana et al., inductive approach of first and second cycle coding [18]. Each idea was assigned a specific “code” with one idea per code. Codes were then grouped when they had the same idea, and higher-level pattern codes (second level codes) were used to organize the data. The in vivo approach was used whenever possible to preserve the phraseology [18]. Theoretical saturation was evident before all FG and KI interviews were fully analyzed, but all data were included.

Once coding was completed, CL started to develop potential themes and worked with HK and RV (researchers on the M2E project, intimately involved in facilitating implementation) to organize the data and categorize emerging themes through an iterative process. Thematic memos were developed which provided a rich description of the theme supported by exemplar quotes and these were revised in an iterative process with RV and HK. Several uncoded transcripts (4-5 transcripts per researcher as selected by CL, total *n* = 13) were reviewed by RV, HK and JB to familiarize them with the sites and data. The four researchers then considered these data when reviewing the emerging themes as exemplified by the thematic memos. Further discussions were held among the researchers until all authors agreed the themes were representative of the data provided in transcripts. Triangulation with other findings, including M2E data and M2E researcher experiences were also used to confirm the themes [19, 20, 22]. JB provided external review since he was familiar with M2E, yet not as connected to the M2E data collection as HK or RV. Member checking of themes was also obtained during a stakeholder meeting with M2E champions and co-investigators (*n* = 25). Further opportunities to confirm the credibility of themes occurred in webinars and conference presentations for acute care clinicians.

### Ethics approval and consent to participate

Ethical approval for M2E was obtained from the University of Waterloo Research Ethics Board (ORE #20590) and from the ethics committees at each of the five participating hospitals (Niagara Health Ethics Board, Ottawa Health Science Network Research Ethics Board, Health Research Ethics Board of the University of Alberta, Regina Qu’Appelle Health Region Research Ethics Board, Concordia Research Ethics Committee). Data collection directly from staff required informed written consent, which was attained prior to data collection. All data remained anonymous to all researchers, excluding CL, and was stored in password-protected files on locked computers. Written consent was taken before each interview or FG, complemented with a verbal reminder before recording began. Participants were aware that some quotations would be used and that these would be de-identified by person and hospital before use.

## Results

**Fig. 1:**
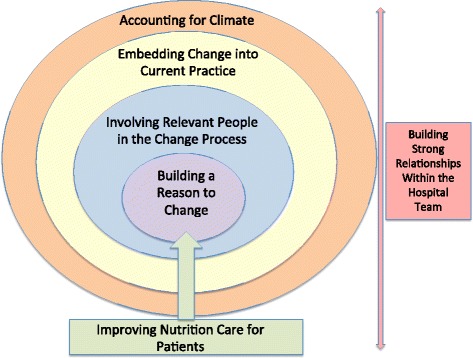
Framework describing the themes regarding making change to nutrition care in the hospital setting

Demographics of participants are included in Table 2. The themes that emerged from this study focused on how to make change to nutrition care practices in the hospital from the perspective of a variety of hospital staff including: registered nurses (RNs), registered dietitians (RDs), physicians, food service workers, management etc. At the core, staff indicated that there needs to be a reason for them to change their practices, and this was typically to benefit the patient. Growing from that reasoning was the need to involve relevant people in the change process and a focus on how to embed change into current practice. Context was key; thus understanding the context and overall climate should be considered when working within the hospital structure. Finally, strong relationships within the hospital teams were seen as vital throughout the change process. A heuristic of these themes is represented in Fig. 1 with details included in Table 3.

**Table 2:** Participant information for all focus group and interviews

Demographic Information		Interviews	Focus Groups
		n (%)	n (%)
# of Participants		40	93
Gender	Female	29 (73%)	79 (85%)
	Male	11 (27%)	9 (10%)
	Missing Data	0	5 (5%)
Age (Range)	<30 years	4 (10%)	28 (30%)
	30-39 years	8 (20%)	21 (23%)
	40-49 years	14 (35%)	17 (18%)
	50-59 years	10 (25%)	17 (18%)
	60+ years	4 (10%)	5 (5%)
	Missing Data	0	5 (5%)
Profession	Dietitian	6	7
	Diet Technician/Diet Assistant	4	2
	Food Service	2	3
	Food Service Supervisor/Manager	7	2
	Dietitian + Food Service	3	0
	Registered Nurse (+Discharge Planner)	7	28 (+2)
	Registered Practical Nurse/Licensed Practical Nurse	0	9
	Nurse Practitioner/Clinical Nurse Specialist	0	1
	Health Care Aide/Personal Support Worker	0	11
	Attending Physician	6	1
	Physiotherapist/Occupational Therapist	0	14
	Speech-Language Pathologist	0	6
	Discharge Planner (+Registered Nurse)	0	1 (+2)
	Management^a^	13	0
	Other	9^b^	8^c^
	Missing	0	5

Note: some participants indicated more than one profession, therefore the profession values will not equate to the total number of participants

^a^Management Positions: Process Improvement Manager, Manager Patient Flow, Director of Nutrition and Food Service, Manager Clinical Nutrition, Manager, Executive Director (*n* = 2), Clinical Site Lead, Program Director, Unit manager, Clinical Care Lead, Clinical Manager, Director of Food and Logistics. (Many managers also put their clinical role so are included twice in the list of professions)

^b^Others (interview): Admin ED, VP Physician and Integrated Health Services (Medicine Service Line)

^c^ Others (focus group): student (*n* = 3), unit clerk, enterostomal therapist, social worker, physicians’ assistant, pharmacist, educator, case manager (*n* = 2)

**Table 3:** Summary of themes and applicable quotes based on the focus groups and interviews

Building a Reason To Change	
Using drivers to change	*If they think it’s affecting patient care, if they think they’ll make the patients better and if they think it’ll make the care more efficient and less expensive, I don’t think it’s a tough sell at all.* [Site B-I2: Attending Physician]
	*What drove this was... it’s one of the competencies for the students is they have to learn, I don’t know if it’s SGA or if they have to learn physical assessment, so we were like, ‘We don’t do this’. We have to be able to teach the students and able to meet their competencies so we better learn it ourselves, which I am so thrilled that I was like yay. It’s more than just* [name of interviewee] *saying so.* [Site A-I7: RD, Dietetics Manager]
Facilitating the change process	*I think if it doesn’t have a lot of meaning for people and there’s no associated actions tied to it so people don’t see it as valuable so I think that’s probably one of the questions that people tend to skip some of the time. If they can see that value I think that would be very helpful in that change management piece.* [Site A-I1: RN, Manager]
	*But it’s numbers. That’s the challenge. You get to the VP level, all they want to talk about is numbers and right now we’re all talking this is a great idea and nobody argues with them. It’s a great idea but until we get some good numbers that we can prove it, then it’s going to be a lot more powerful then.* [Site B-I7: Senior Management]
	*Simple, effective, with a clear meaningful impact then it’ll be fine. This* [nutrition screening with CNST] *is an easy one. This is not adding an extra 45, you know, we get asked to do, you need to do this now when you’re discharging a patient and it’s actually 40 min for every discharge and we’re like, whoa, you just increased my day by two hours. So that’s hard to sell. *[Site B-I2: Attending Physician]
Being ready for change	*I think when you talk honestly and you talk openly about* [the change] *to them and you tell them right off the bat we don’t promise to have all of the answers. We don’t promise to know everything but we’re going to work with you and we’re going to figure it out as we go, right? I think the thing is, is we’ve been talking about it and we’ve done other changes and they’ve seen how we’ve proceeded to do those other changes and we’ve done them exactly how we’ve said is that we have to start somewhere. Here’s where we’re starting. We’ve taken two or three weeks where we’ve tweaked them and made changes. We’ve listened to their comments and suggestions and then we’ve improved it.* [Site B-I3: Food Service Manager]
	*… when they start balking the system and not wanting to change, the thing that we always remind them is that, do you have a cell phone? Do you have an iPhone? “Yes.” How many times have you updated your iPhone in the last year? “Well three or four.” Then why is your work not the same? And I think if you put it into those terms, that speaks to every single one of them. They say, “Oh yeah, that makes sense.” *[Site B-I3: RD, Manager]
Involving Relevant People in the Change Process	
Involving staff in the change process	*It’s almost like saying every patient needs to walk but that doesn’t mean that physio needs to walk with every patient. Right. Every patient needs proper nutrition care but that doesn’t mean it should necessarily be a dietitian.* [Site E-FG2: Physiotherapist]
	*I think it’s really important to get down to that front level staff so they understand what the process and what the impact might be but also that they also have an impact as to how it’s going to be rolled out and positive, how those interactions are going to be played out.* [Site 5, Int 3: Food Service Manager]
	*Getting feedback from those involved. Whenever I roll out change with my staff, I always get their feedback because they’re so knowledgeable; because they’re the ones actually doing it.* [Site C-I3: Food Service Supervisor]
Involving patients, families and friends in the change process	*A large group that would be good to involve is the patients, and or the families. … They’re sitting here for long periods of time with nothing to do. If they, if we have some way of involving them, I think. And if they understood, because it’s the families who have to sustain whatever plans we put into place when they leave here.* [Site B-FG1]
Involving volunteers	*We would love to use volunteers. … that would be wonderful to have them on the unit because at mealtimes, because then they can go in and visit the patients and get them the assistance. Those that don’t need to be fed they can take care of setting them up or maybe helping setting the trays prior to us getting there; that kind of thing. We would love to see volunteers.* [Site C-I3: Food Service Supervisor]
Obtaining buy-in from stakeholders	*They need to understand why they’re doing it and then I always think personalizing it to the client or patient that usually is a pretty good sell. Then I think people will buy in and we could get some sustainability.* [Site A-I12: Manager]
Embedding change into current practice	
Incorporating small changes slowly	*So you have to start small, iron out the kinks if you will and then replicate it as you can if humanly possible so.* [Site A-I12: Senior Management]
	*I certainly think that people feel a lot less, I think, angst knowing that they’re trialing something for a short period of time and of it is not going to work out we can tweak it and modify it and that it’s not something that’s for, you know longer periods of time.* [Site E-I3: RD, Clinical Site Lead]
Benefiting from existing structures and processes	*… what I can offer is looking at ways of reducing a length of stay by designing systems… how do I connect the process and identify these patients early on so that the discussion, the conversation can happen earlier on a lead time is always money. How I would try to embed this process? … How I do embed it would be…there would be a way of identifying them right off the bat, upon admission on our board.* [Site D-I3: Manager]
Accounting for staff perceptions of best practice	*…when and how we roll this out if we can involve the staff as much as we can to bring them into it, the more they play a part in the pre-rollout the more successful we’ll be.* [Site B-I5: Clinical Manager]
	*Yeah, give examples, maybe give some concrete patient examples that they can see that relate to medicine.* [Site B-I1: RN]
Facilitating the integration of sustainable change	*So it has to be standardized, right, and it has to be there all the time so, yeah. And part of the problem is there’s, you know you’re going to have this problem on a ward or - we have patients all scattered throughout the hospital and this ward sometimes has non-medicine patients on it so you have to pick your audience and decide what you want to do. It’s totally doable.* [Site B-I2: Attending Physician]
Accounting for Climate	
Working within the constraints of the hospital structure	*… we had to bring more hours back into the department because some of those hours were with housekeeping. … got involved with the union, reallocating hours, job re-assignments, redevelopment of job routines. There was a lot involved with that. Summary training, because new employees coming in maybe didn’t do tray delivery so we had to retrain. There’s a lot involved with that.* [Site C-I3: Food Service Supervisor]
Presenting nutrition as a benefit or value to the hospital	*Nursing to patient ratio’s gotten lower and lower, higher and higher, lower and lower. Patient and nurse ratio has gotten higher and also can’t afford to make it lower. It’s bad care. No one says it corporately but we all know it. Even the hospital says they’re firing 57 nurses and then* [name] *gets on the radio and says, “But it won’t affect any patient care.” Come on.* [Site B-I2: Attending Physician]
	*I think that we’re pretty engaged. As a health region we’re engaged and again I think that’s one of the benefits of having a smaller health region is initiatives like this can gain a lot of momentum and be shared because they’re interdisciplinary, they cross so many different areas and we’ve had lots of opportunity to talk about it.* [Site A-I6: RD, Manager]
Building strong relationships within the hospital team	
Using the right amount of communication with the right message	*I think that one of the keys if we want to make sure that this is something that’s well known and people can anticipate potentially being replicated, is to do a good amount of communication. So not over-communicating but making sure that it at least stays in the forefront of peoples’ minds and I don’t think we should isolate that just to one group because I know that a more senior leadership level or the people that are directly involved.* [Site A-I12: Senior Management]
Developing and maintaining trust	*Feeling comfortable enough to know who to ask and knowing that it’s going to happen. … And I think the relationship, like KE1 and I, the CCL and I have with our staff is that they’re very comfortable to come and tell us what they need and how they feel.* [Site B-I5: Clinical Manager]
Engaging the team	*Our group has met several times so we obviously feel comfortable as a group but actually working together on behaviour change and the PDSA cycles and all that.* [Site E-I3: RD, Clinical Manager]
Breaking down individual silos	*I like to see allied help because I’m a nurse; my background is nursing. I really like to the allied members of the health team engage the nursing side of it, because so often we’re so siloed in our specialties that we don’t come together.* [Site C-I1: RN]
Using communication tools	*I have a communication book in my department. If I’m making departmental changes, I always leave them there. I hold huddle meetings when I’m here on site. … I try to bring people together to go over the issues and the communication book to reach staff that I don’t see. Then if it’s a huge impact that needs to happen right away, I will call staff even at home and say, “This is changing immediately. This is what’s happening.” This is what I try to do. *[Site C-I3: Food Service Supervisor]
Using face to face communication	*Just speaking from the change management project that we work with, it was a really interesting experience; first for myself on that level for having that many people around that table representing different areas that are touched by nutrition services. I was pleasantly surprised at the input and the feedback from everybody but equally as much surprised that through the discussion there was a lot of aha moments for people.* [Site E-I3: RD, Manager]
	*How we can improve communication ... We did a walk around. We met with* [name] *the manager, found ways to identify to nursing staff whether a patient ate less than half of their tray. We did some brainstorming.* [Site A-I5: RD, Food Service Manager]

### Improving nutrition Care for Patients

To encourage thought development during interviews, participants were asked about what their unit/hospital was doing well regarding nutrition care and what improvements were needed. Answers focused on the need for improvements to patient-centered care, protecting mealtimes, and mechanisms for making sure food was available and accessible to patients.

Participants described the need to provide patient-centered care that focused on the whole person and their individual needs. This philosophy of care was about getting back to basics: “*recognizing that it’s not just about the task that you have to do in front of you but also both the patient’s whole well-being and nutrition”* [Site A-I4: RN, Nursing Management]. Using food intake to understand the overall needs of the patient was also noted; “*That* [what is left on their meal tray] *tells you everything about their functional status or their mental status or whatever”* [Site B-I2: Attending Physician]. The provision of eating assistance was also mentioned as a way to understand a patient: *“And that’s it’s not just feeding them but there’s a really good time to assess them as well for different pieces there in terms of their nutrition…”* [Site A-I4: RN and Nursing Management]. Thus food and its capacity to centre care in a more person-centred way was a key reason to improve the nutrition practices in hospitals voiced by participants.

Ways to protect mealtimes were also discussed as a key way to improve nutrition care. “Protecting mealtimes” was described as: decreasing interruptions by planning a routine, and ensuring that food was available/accessible. One site was particularly concerned with meal timing and the effect it had on the patient experience and food intake:

*If we can say to patients, ‘Meals will be delivered within this 20-minute window, have your family come and help you’, but right now we can say meals are delivered at noon. Well their family might come at noon and lunch is delivered at 11:20 so it’s sitting there cold and the patient doesn’t want to eat it anymore.* [Site A-I7: RD, Dietetics Manager].

The availability and access of food was a frequent point of discussion including: having food available on the unit; making sure patients are set up to eat; clearing the patient area so the tray was within reach and not surrounded by unappealing items or smells: “*It’s just the environment isn’t inviting and the commode is right beside the bedside”* [Site A-FG3]; providing encouragement for patients to eat; identifying those in need of eating assistance; making sure packages are opened; decreasing staff breaks during patient mealtimes; and when applicable, accommodating food from outside the hospital. Outside food can be accommodated by: “*Make*[ing] *room for the families to bring in their food”* [Site E-FG1]. Challenges in food delivery for isolation patients were noted including that the food may get cold or the meal totally missed for the patient. Recommendation for improvement were also give, for example: “*We* [now] *leave the* [isolation patient] *meals at the nurses’ desk”* [Site C-I3: Food Service Supervisor] as a way of reminding the nurse that the tray needed to be delivered to the isolation patient. There was also concern regarding lack of clear communication about NPO status (nothing by mouth). As noted by a food service team member: “*They’re* [food service staff] *fearful of handing out a tray to an NPO patient because it could delay surgeries or have a significant impact on a lot of different things by feeding a patient.”* [Site C-I3: Food Service Supervisor]. The discussion regarding these specific elements of nutrition care was used as a mechanism to encourage discussion regarding how changes are made within the unit/hospital.

#### Building a reason to change

At its core, hospital staff need a reason to change their practice before embarking on a change effort. Key improvements described by sites were presented above. In addition to these specific desired changes, participants described benefiting the patient as a key driver for change as well as organizational priorities. Staff and management had to see the change as valued and important, while considering their current context and what was feasible. Participants also described practical ways for building a reason to change and several facilitators were offered. Finally, it was noted that determining and building capacity for change was foundational before any implementation efforts could begin.

Participants described a variety of drivers or reasons for making change of which the benefit to the patient was most salient: “*I’m up for trying anything as long as it’s for benefit for our patients.”* [Site B-I5: Manager]. Other drivers were organizational requirements that led to efficiencies. It was noted that timely discharge from the hospital was a key organizational driver for practice change, as was the need to meet student requirements for internship placements, or other regional-level requirements. As noted in this FG, malnutrition could be described as a “barrier to discharge” which raised hospital costs and effected patient flow to help prioritize the issue and make change:

*Identify it* [malnutrition] *as a risk to “barrier to discharge”, because they* [patients] *are not eating nutritiously, they’re not healing as quickly as they possibly could be, therefore their discharge is delayed. That makes people pay attention.* [Site B-FG2]

In addition to patient benefit and organizational drivers, other facilitators to change described by participants included: linking the change to a valued action, keeping the plan simple, and proving the change was worthwhile. For example, if screening led to the RD seeing a patient sooner and addressing nutrition and food concerns, this could be seen as valuable by nursing, potentially minimizing challenges later in the hospital stay. As noted by this manager, meaningfulness of the changed behaviours was key:

*I think if it doesn’t have a lot of meaning for people and there’s no associated actions tied to it, people don’t see it as valuable … If they can see that value I think that would be very helpful.* [Site A-I1: RN, Manager]

Keeping the messaging simple as to what needs to change also supported this step in the change effort, as did continually educating the staff about the issue and what they needed to do. It was also described that enlisting ‘believers’ in the issue early on could be one factor in building a reason to change. These ambassadors within various disciplinary groups could help spread awareness beyond those championing the effort.

*… it’s a cultural transformation so like any other cultural transformation you need to start with the believers first. Get that out of the way and then work on the people that are either resisting change or taking a longer time to change.* [Site D-I3: Manager]

Further, change needs to be visible on the unit and the ideas need to be marketed in a way that encourages and supports the change.

*It’s got to be like hands on. It’s got to be – it’s got to be people that are visible on the floor to see what goes on; not just me reporting it or the charge reporting it. … they’ve got to see it.* [Site C-I5: Diet Technician/Diet Assistant]

By being evident through personal experience of all staff on the unit, a change was more likely to be seen as worthwhile and thus perpetuated its continuation.

Several practical ways of building a reason to change were also described. For example, data supporting the need to change a process could be used to make the argument for the necessity of improvements, such as malnourished patients being missed because there was no screening process in place. Using their own local data and comparing to a standard to show deficits in practice was an example provided:

*… as with anything there’s going to have to be that audit-review-feedback loop that is built in so that staff understand that … it* [nutrition] *is important and hopefully catch it before the patient is discharged or make sure it’s corrected for the next patient*. [Site A-I2: Senior Management]

Other practical ways of building a reason to change were to continually educate people about why the change is important, and using short sessions such as huddles or in-services. Reminders, such as posters, were also considered important tools to keep staff engaged and informed.

Finally, building a reason to change also included developing capacity in a variety of ways. Hospital staff highlighted that they needed to be ready to make the change, that change efforts had to be realistic and that the change process had to be normalized. As described by this informant, change was a constant in the hospital and a strong foundation for accepting and making change was required:

*It doesn’t matter what people want to do, if you don’t have the right foundation set, you’re going to lose things, so that’s the point of it. ... Healthcare is liking boxing an octopus, you can’t put two hands up there’s a lot of other things coming at you so the more you try to predict all these little variables, diets, homecare all that…* [Site D-I3: Manager]

Staff need a reason to change their practice, and should be supported to do so through changes that are feasible and show clear benefits, particularly to the patients.

### Involving relevant people in the change process

When making change, participants discussed needing to have the right people involved at the right level at the right time. Discussions highlighted that everyone (management, front line staff, food service, allied health, patients, families, friends, volunteers etc.) should have a clear understanding of their role in the change process (improvement of nutrition care) and be brought in at the appropriate time. Departmental silos were a key issue that needed to be addressed, as well as building ownership of nutrition care, particularly mealtimes, rather than deferring accountability. Volunteers, family and patients were noted as being part of the change process by having specific roles and understanding expectations.

All sites discussed the challenges, yet importance, of involving relevant people in the change process. Staff are busy and clinical commitments take priority. Several participants mentioned departmental silos, and with nutrition being relevant across departments, there was a desire to find a way to overcome these silos and have everyone working together. For example, a food service manager discussed her desire and attempts to encourage food service to be treated as part of the unit team, yet often felt her team was excluded. One attempt to overcome this silo was the piloting of a model where the same food service worker delivered and picked up the tray:

*Hopefully … that staff member will become part of the team upstairs but it’s very much still hire-keep where the support staff, and nutrition belongs to that group, are viewed down on and so it’s trying to convey the message that, “you know what? You guys* [food service staff] *play just as an important role as everybody else. Everybody has a role to play and it’s different. Mine is different, theirs’ is different and it’s engaging the staff and making them realize the importance that they do too.”* [Site B-I3: Food Service Manager]

When discussing the involvement of the relevant people, the need for accountability was also mentioned. Accountability was discussed regarding involvement in the change process and following through on designated tasks, as well as in the overall current lack of accountability for meal times: “*There’s absolutely no one who’s accountable for mealtimes.*” [Site A-I7: RD, Dietetics Manager]. Reasons for this lack of accountability were discussed, and the suggestion was made for how to think of this as everyone’s responsibility: *“It’s almost like saying every patient needs to walk but that doesn’t mean that physio needs to walk with every patient. Right. Every patient needs proper nutrition care but that doesn’t mean it should necessarily be a dietitian.”* [Site E-FG2, Physiotherapist]. Clarity regarding the responsibility of each staff member was discussed as a method for increasing accountability. Opinions were mixed regarding whether specific tasks should be designated or if everyone should be encouraged to participate in nutrition care.

All sites discussed the potential value of volunteers having a role and supporting nutrition care: “*We would love to use volunteers.”* [Site C-I3: Food Service Supervisor]. Recruitment challenges were highlighted, as well as capacity and comfort level of the volunteer. “*I think the biggest challenge was just filling that* [volunteer] *position all the time.”* [Site D-I4: Food Service Manager]. An area of concern from both staff and volunteers was about providing eating assistance to patients. When eating assistance was removed from the required activities, staff and volunteers were both more comfortable, which also facilitated volunteer recruitment.

Although all sites mentioned changes being for the benefit of the patient, only a few sites mentioned the role of patients, families and friends in the change process. For example, when discussing ways to decrease mealtime interruptions, one RN wanted to find out what patients thought about mealtime interruptions and whether they would rather be interrupted for a test, or have uninterrupted mealtimes. Expectations and perspective of the patients, family and friends needs to be considered when developing an improvement plan.

Diverse stakeholders need to be involved in the change process at various points and their buy-in for change is essential. To obtain buy-in, the justification should be personalized and the need for the change should be clearly visible to the group and individual. “*They* [stakeholders] *need to understand why they’re doing it and then I always think personalizing it to the client or patient that usually is a pretty good sell.”* [Site A-I12: Senior Management].

### Embedding change into current practice

To make changes last, they need to become embedded into current practice. To promote sustainability, participants mentioned that changes should be small and start slowly. The benefits of embedding the changes into existing structures and processes were discussed as ways to decrease the change burden and increase likelihood of a lasting change. Participants mentioned that opinions and perceptions of staff regarding best practice and ways to embed the change needs to be considered, recognizing that opinions may not always match reality or best practice. Yet, it is important to make sure staff opinion is incorporated into the change process to build ownership. Further, to facilitate the integration of sustainable change, the process needs to become part of the routine and be supported by existing processes and evaluation methods.

When embedding change, there is a need for changes to start small, yet have potential for large impact. “*What I’m hoping is that people will identify* [in M2E] *some simple small changes that will have a maximum impact for the patient.”* [Site E-I6: Senior Management]. Short pilots were seen as a way to test ideas that can be evaluated, modified and re-trialed. “*So you have to start small, iron out the kinks if you will, and then replicate it as* [much as] *you can, if humanly possible.*” [Site A-I12: Senior Management]. Results throughout the change process should be fed back to the staff involved so they can gauge their progress. One RD identified how they planned to embed the subjective global assessment (SGA; the key nutrition assessment tool in INPAC) into current practice:

*… the expectation was that we would learn the basic idea of it* [SGA] *and slowly start to incorporate it in our daily routine. We all agreed on sort of a minimal number of times we would use it say per day and we slowly built that into people’s work routines as they felt more comfortable and became more skilled at using it.* [Site E-I3: RD, Manager]

Not all changes need to be new initiatives. Many participants mentioned the benefit of using and adapting existing structures and clinical governance processes. For example, all sites discussed embedding nutrition screening into current admission forms. “*…it sounds like it’s* [CNST] *going to be integrated into an already existing process … I think that’s helpful as opposed to making it a separate process.*” [Site A-I1: RN, Manager]. Other current structures that could support embedding of a new practice include changing the role of food service workers so they can be considered part of the unit team, using existing quality improvement teams to support the changes, and tweaking whiteboard systems (a method used in hospital to track patient progress including which specialisms need to see the patient before discharge) to incorporate nutrition care activities.

To embed change, it is important to understand staff perceptions and to discuss further when their perceptions do not match the local evidence. Providing education using local data as well as evidence for why the change is needed can help to shift perceptions. For example, misperceptions with respect to standard nutrition care practices that reduced barriers and supported food intake for all patients (i.e. setting up patients for their meal) were noted. There were mixed opinions between interviews in the same sites and even within the same interview, regarding whether nutrition care practices were adequate. For example, one participant indicated patients were always ready for the meal, yet later admitted there was not enough time to get everyone ready and more support was required. One RD felt that the reality of standard nutrition care differed from the staff perception. *“I think people think that they’re doing better than they’re doing. I think people try and have a good heart but the reality is different than what the perception is.”* [Site A-I7: RD, Dietetics Manager]. Local data tracking these care activities can help to align varying staff perceptions with reality and demonstrate a need for change.

Staff are the experts regarding their daily routine and need to be consulted if changes are expected to impact them. “*The more they* [staff] *play a part in the pre-rollout the more successful we’ll be.”* [Site B-I5: Manager]. Several strategies were discussed regarding how to bring staff on board, particularly when their perceptions did not match best practice or local evidence. For example, in one site, it was indicated that front line staff had inaccurate information about the food and most staff had not tasted it themselves. “*…Generally, I think staff don’t find it* [hospital food] *appealing and I think the patients won’t if the staff portray that.”* [Site E-I5: RN, Unit Manager]. Food service found it frustrating that these staff were encouraging this negative attitude with patients, yet staff did not understand the sourcing (it was local food), production (how the food arrives at the hospital), or even the taste of the food being served. The approach to address this perception was to have management and staff taste the food, along with reminders about the local sourcing and the diet order process. Thus, personal experience is also needed to embed a change.

Other facilitators to embedding change focused on standardizing the process and re-evaluating throughout the embedding stages so there would be an understanding of what change has occurred and what seems to have been embedded into practice. “*It has to be standardized, right, and it has to be there all the time...”* [Site B-I2: Attending Physician]. For example, when getting screening started, auditing and reporting the screening rates was considered an important way to embed practice. Data could then be followed with discussions with front line staff regarding what further improvements could be made to embed the practice into routine.

### Accounting for climate

Typically context is key and an overarching element to consider, however these discussions went beyond context, to touch upon the overall climate. Climate focuses on the values of the organisation, including the means, motivation and opportunities for innovation [16]. These values can include the values of the hospital and larger health region, including current policies and regulating bodies. Many participants discussed the need to work within the constraints of the hospital structure, including the requirements of the food service delivery mechanisms, and the regulations of the health region when considering a new practice. To work through these limiting factors, participants highlighted how improved nutrition care needs to be presented as a benefit or value to the hospital, focusing on saving money and engaging the greater system. *“If they think it’ll make the care more efficient and less expensive, I don’t think it’s a tough sell at all.*” [Site B-I2: Attending Physician].

To work within the given hospital structure, participants discussed the need to navigate complicated processes. One small change might have many different elements that need to be accounted for, such as the inflexibility of the food production and delivery processes as food is typically made and usually plated offsite. Other complicated processes included changing staff roles, routines, hiring, unions etc. which all need to be recognized and considered in an attempt to minimize delays or barriers within the change process. Regarding hospital policy, participants mentioned the need to work within the current policies and work towards improvement when there are gaps. *“Ultimately to have the policy set up so that it becomes a policy within our organization that this is what we do.”* [Site C-I3: Food Service Supervisor].

Improvements in the current nutrition care needs to be presented as a benefit to the hospital from a variety of perspectives that account for the current climate. Hospitals are under pressure to have policies that encourage patient-centered care and save money. Change drivers or champions should present the case that prioritizing nutrition is one way to address both patient-centered care and introduce cost savings. This requirement for a change in practice and ways to save money were addressed, including highlighting evidence that malnourished patients stay longer and cost more. Another strategy was to find ways to benefit the bottom line, such as decreasing waste. *“*[We need to] *have a bit more of a resource savvy way of going about doing some of those things because there’s huge financial impact to all that waste.”* [Site A-I9: Manager].

### Building strong relationships within hospital teams

An overarching aspect in these discussions was the need for strong relationships, which is considered an underlying concept within all other themes in order for change to be effective. Strong relationships are built on good communication, trust and team engagement. Participants emphasized the need to use the right amount of communication with the right message, as well as the importance of developing and maintaining trust. Many participants discussed the inefficiencies created by departmental silos and ways for this to be overcome. Team engagement in the issue and building an attitude that we ‘are all in it together’ was a way to build relationships. Specific strategies for building strong relationships focused on use of communication tools and the importance of face-to-face communication.

Discussions highlighted the need for the right balance of communication where people are aware of the change but are not overloaded. *“… you have to find a way to do that* [educate them] *without inundating people so they see beyond it.”* [Site C-I1: RN]. The message should also indicate that the change *must* happen rather than *might* or *should* happen.

*When she first rolled it out it was more about a ‘nice to have’ not a ‘must have’. It was a “Wouldn’t it be nice if we could?” It was almost built-in optionally. Where our tact this time will be much different. It’ll be more about we will have an expectation that you’ll have the table cleaned. We will have an expectation that your patient’s sitting up and ready to eat.* [Site B-I7: Food Service Manager]

One manager discussed how his team was effective because they had strong communication skills and teamwork. Front line staff trusted they could approach management with a concern, and whenever possible, management would address that concern.

*Feeling comfortable enough to know who to ask and knowing that it’s going to happen. … And I think the relationship, …* [we] *have with our staff is that they’re very comfortable to come and tell us what they need and how they feel.* [Site B-I5: Clinical Manager]

Across all sites, engagement was discussed as an important component within the change process. Lack of engagement from the relevant people in the process was often mentioned as a reason that a project lacked sustained impact.

*To me the biggest, I guess issue, … is lack of engagement. People need to understand why you’re doing it and they need to, if not agree, at least see the benefit and if you can get – because we need everybody. … if we can communicate properly to them and give them the information that they need and show them the why you’re doing it, right?* [Site A-I12: Senior Management]

A lack of communication across departments and individuals was also described. In several interviews, a problem was highlighted in one discussion and the solution was mentioned in the next. Unfortunately, many staff were not aware that the solution already existed and so it was not in regular use. Participants highlighted this problem by discussing the individual silos and the need to improve communication channels. When changes do happen, staff should be aware of those changes and be able to use it to their benefit. A lot of effort is wasted if a change is made yet never used because staff were not aware or consulted.

There was a need to recognize the role of other individuals and how they can work together as a team to improve communication, and in turn, impact patient care. “*I really like to see the allied members of the health team engage the nursing side of it, because so often we’re so siloed in our specialties that we don’t come together.”* [Site C-I1: RN]. Talking to people directly, face-to-face when possible, was mentioned as one strategy for improved, clear communication and the building of stronger relationships. Group discussions, such as the FG to collect this data, were said to be informative and provided a beneficial contribution to the change process. “*I’m finding this* [the FG] *very educational. If we can do something like this, even once every 6 months, or something where we’re all sitting down and saying what are the issues, how can we do this better.*” [Site E-FG1: Physicians’ Assistant].

## Discussion

Hospitals are unique locations, where clinical commitments to patients are the priority. However, the clinical importance of adequate nutrition care and its impact on patient-centred care is recognized but not always acted upon [14, 15, 25, 26]. Raising awareness and providing education about the issue is important, but it is not enough. Hospital processes and systems need to be adapted and strong relationships built with clear channels of communication, so that improvements can become embedded into routine [13]. For improving nutrition care practices, dietitians cannot do this alone. Dietitians should work as part of an interdisciplinary team to effect beneficial changes in nutrition care for all patients [15].

The Nutrition Care Model (NCM) is designed to visually represent the American Dietetic Association’s, Nutrition Care Process (NCP) [27]. The NCM focuses on the role of the dietitian and interaction with a patient, while working within a larger system. Within this model, in practice, most of the focus regarding improving nutrition care has been at the individual patient level, focusing on the middle four sections of the circle, including assessment, diagnosis, intervention, monitoring and evaluation. The results of this study indicate that in order to make change to nutrition care in a hospital setting, more focus should be placed on the outer layers of the circle, and thus the larger hospital system. Communication and collaboration are key when trying to improve practice. A prior study implemented the NCM practice of dietitians charting with standardized terminology as a pilot in two hospitals [28]. Authors recognized that change takes time and requires a variety of strategies including education, feedback, reminders and positive encouragement. The dietitians were most affected by this change in practice, however it appears that little focus was paid regarding the existing climate, determining whether the dietitians were ready for the change, whether other members of the clinical team were informed of the change or about how it would impact their practice. Even within these two hospital NCM pilots, the context and strategies used were different, emphasising the need to look beyond raising awareness or knowledge when changing practice, and also the need to consider the climate, or values, of the organisation [27].

An article by Leggat and Dwyer, focusing on improving hospital performance, strongly emphasised the need for “good people management” and the impact that this can have on culture change [28]. This emphasis is in line with themes regarding building strong relationships, working as part of a team, and begins to touch upon considering the climate, in order to facilitate the change process or innovation described in the current work. Climate is a broad concept that is difficult to articulate, often misused, and often overlooked during implementation [16]. However, positive climate has the potential to significantly impact change, as it includes policies and practices that encourage means, motives and opportunities for innovation and change [16]. It is encouraging to see these interview/FG discussions incorporate aspects of climate and recognise the overall impact that it can have on the success of a change or innovation.

Behaviour change strategies within acute care need to be considered during change processes. A review of reviews looking at professional behaviour change in healthcare found that types of interventions could be split into three main categories: persuasive; educational and informational; and action and monitoring (audits, reminders, education etc.) [29]. These types of interventions are in line with the findings of this study, and are consistent with the Michie et al. Theory of Behaviour Change and the Behaviour Change Wheel (BCW) [30]. The BCW highlights aspects to be considered when designing behaviour change interventions [24], specifically the “sources of behaviour” including capability, opportunity and motivation (COM-B). The BCW was considered when conducting the M2E interviews and FG, and it is recommended that it be consulted during any change or implementation process.

Research on how to implement clinical guidelines in acute care and the findings from this study are consistent, however few studies focus on perspectives from a wide variety of staff/professions and many studies only focus on nurses. A systematic review of nursing interventions designed to normalize implementation of clinical guidelines highlighted the need to: integrate the change into the current workflow; involve and engage the relevant communities of practice and recognize the reason for that engagement; and build shared commitments across professional boundaries [31]. Another study interviewed nurses to examine factors that facilitate the effective implementation of clinical guidelines [32]. It was noted by these authors that all staff should be involved in the implementation process; continuous feedback loops should be used; and the change had to be seen as beneficial, balancing priorities and cost [27]. Although there are similarities within the themes and the current study, our research focuses on the perspectives of many hospital staff and management, going beyond the ideas of a single profession. Interprofessional perspectives are needed to overcome departmental silos. As discussed above, few studies emphasize the importance of the overall climate, which as noted in this study, extends beyond priorities and cost, and includes the overarching values of the hospital.

An additional learning point applicable to practice, was that although the FG were designed for data collection, they ended up being used as a way to engage M2E unit staff prior to implementation of INPAC. It was suggested by several participants involved, that having discussion groups throughout the change process would be helpful to increase staff awareness and engagement. These discussions may be an opportunity to bring staff on board, to include their opinions and further engage them in the change effort. In M2E, short summaries of these results were provided to each site after the site visit so they could consider the staff perspectives during INPAC implementation.

### Strengths and limitations

The aim and strength of this study was that it included a variety of perspectives from hospital staff and management, which supported the emphasis on an interdisciplinary approach to nutrition care. Previous research has generally focused on perspectives of individual healthcare professionals, particularly nurses. Canvassing opinions more broadly (for example patients, families, volunteers) would have provided additional depth, and a more comprehensive look at the overall hospital structure, beyond the views of staff and management. This was beyond scope of the current study, and is considered worthy of further exploration.

Another strength is that a large number of interviews were collected and data saturation across themes was achieved early in analysis. An a priori target for sample size of 3-4 KI interviews and 2 FG per site (15-20 interviews and 10 FG total) was provided to sites and deemed suitable. However, each champion recruited more than the target KIs as there was a desire to represent staff and management perspectives more broadly. All interviews were pre-arranged, with most conducted during the 2-day site visit, and all scheduled interviewed were completed. Due to the quality of data and saturation of themes, no additional or repeat interviews were indicated. As champions selected the interviewees, it was not possible to record how many refused to be interviewed. It was also not possible during the FG to distinguish between those who were unable to attend due to clinical commitments, compared to those who refused to participate. In FG, a M2E champion or research associate was present to take notes. Although FG participates were reminded the conversation remained confidential, the presence of this individual may have influenced the participation or discussion.

Another limitation is that data was not analysed by profession or by site. As the context varied across the five sites, new ideas were observed across all sites prior to reaching saturation, however similar messages were seen throughout data collection which reinforced the approach of looking at all sites and professions as one. This combined approach also encourages and reflects the interdisciplinary approach of implementation and data collection.

Due to the volume of interview data, FG were not sent for transcription, however detailed notes were taken by listening to the audio-recorded discussion, and key sections (i.e. exemplar quotes) transcribed verbatim by CL. Data was not collected with the intention of being generalizable. Yet, the similarity of findings across these five sites increases the external validity of the results. To demonstrate credibility and trustworthiness, methods and results are described in detail, with quotations and additional data presented in table format [18]. Another limitation is that transcripts were not returned to all KIs for member checking or for further clarification. However, summaries were sent back to each hospital for comment and clarification shortly after data collection to ensure that key components were consistent with their perceptions. In some cases, these summaries were reviewed by the KIs. The final themes were also discussed with the champions and co-investigators to confirm that the themes resonated with their experience and were further presented in webinars and conferences.

## Conclusion

Hospital staff need a reason to change their nutrition care practices and a significant change driver is patient benefit. Dietitians can facilitate the process by championing the change and working with an interdisciplinary team to provide more comprehensive nutrition care across disciplines. All relevant stakeholders need to be involved to embed change into the current system. Climate, describing the overall values of the hospital, should be considered, as it is an influencing factor in all changes. Change is difficult but achievable and strong relationships within the hospital and teams are important when working towards changing practice.

## Abbreviations

BCW: Behaviour change wheel
CNST: Canadian nutrition screening tool
COM-B: Capability, opportunity and motivation
FG: Focus Group
INPAC: Integrated nutrition pathway for acute care
KI: Key informant
M2E: More-2-Eat
NCM: Nutrition Care Model
NCP: Nutrition care process
RD: Registered dietitians
RN: Registered nurses
SGA: Subjective global assessment
